# Nursing and society: Evolution of Nursing and of capitalism in the 200 years of Florence Nightingale*

**DOI:** 10.1590/1518-8345.4482.3425

**Published:** 2021-05-21

**Authors:** Rodrigo Nogueira da Silva, Mrcia de Assuno Ferreira

**Affiliations:** 1 Universidade Federal do Rio de Janeiro, Escola de Enfermagem Anna Nery, Rio de Janeiro, RJ, Brazil.

**Keywords:** Capitalism, Nursing Care, Health Economics, Health Care Sector, Nursing, History of Nursing, Capitalismo, Cuidados de Enfermagem, Economia da Sade, Enfermagem, Histria da Enfermagem, Setor de Assistncia Sade, Capitalismo, Atencin de Enfermera, Economa de la Salud, Enfermera, Historia de la Enfermera, Sector de Atencin de Salud

## Abstract

**Objective:**

to analyze the relationships between the development of the Nursing labor and of capitalism over the 200 years of Florence Nightingale.

**Method:**

a logical-reflective and theoretical exposition based on interpretations of historical facts and Marxist theories. The analysis categories were the following: the creation and expansion of the Nightingalean Nursing Teaching System; the subsumption of the Nursing labor to capital; imperialism and international health; and the flexibilization of the Nursing labor.

**Results:**

the expansion of the Nightingale Teaching System has trained nurses on a global scale. The capitalist system transformed the Nursing labor in the twentieth century, culminating in the twenty-first century with precarious and intense turnover of nurses in their jobs.

**Conclusion:**

the Nursing labor, made professional by Nightingale, has assumed in the last 200 years a dialectical relationship with capitalism in which it both determines and is determined by it. New challenges, such as the Industry 4.0 technologies, are constantly imposed on the profession.

## Abstract (pt)

**Objetivo:**

analisar as relaes entre o desenvolvimento do trabalho assistencial de Enfermagem e do capitalismo ao longo dos 200 anos de Florence Nightingale.

**Mtodo:**

exposio lgico-reflexiva e terica baseada em interpretaes de fatos histricos e teorias marxistas. As categorias de anlise foram: a criao e a expanso do Sistema Nightingaleano de Ensino de Enfermagem; a subsuno do trabalho assistencial de Enfermagem ao capital; o imperialismo e a sade internacional; e a flexibilizao do trabalho assistencial de Enfermagem.

**Resultados:**

a expanso do Sistema Nightingale de ensino formou enfermeiras em escala global. O sistema capitalista transformou o trabalho assistencial de enfermagem no sculo XX, culminando no sculo XXI com precarizao e rotatividade intensa de enfermeiras em seus postos de trabalho.

**Concluso:**

o trabalho assistencial de Enfermagem, tornado profissional por Nightingale, assumiu nos ltimos 200 anos uma relao dialtica com o capitalismo em que aquele tanto o determina quanto por este determinado. Novos desafios, como as tecnologias da Indstria 4.0, impem-se constantemente profisso.

## Abstract (es)

**Objetivo:**

analizar la relacin entre el desarrollo de la labor asistencial de Enfermera y el capitalismo a lo largo de los 200 aos de Florence Nightingale.

**Mtodo:**

exposicin lgico-reflexiva y terica basada en interpretaciones de hechos histricos y teoras marxistas. Las categoras de anlisis fueron: la creacin y expansin del Sistema Nightingaleano de Enseanza de Enfermera; la subsuncin de los cuidados de Enfermera al capital; imperialismo y salud internacional; y flexibilizacin de la labor asistencial de Enfermera.

**Resultados:**

la expansin del Sistema Nightingaleano de Enseanza capacit a enfermeras a escala global. El sistema capitalista transform la labor asistencial de Enfermera en el siglo XX, culminando en el siglo XXI con la precariedad y la intensa rotacin de enfermeras en sus puestos de trabajo.

**Conclusin:**

la labor asistencial de Enfermera, profesionalizada por Nightingale, ha asumido en los ltimos 200 aos una relacin dialctica con el capitalismo en la que lo determina y es determinado por l. Nuevos desafos, como las tecnologas de la Industria 4.0, se imponen constantemente a la profesin.

## Introduction

Florence Nightingale was born in Florence, present-day Italy, in 1820, into a wealthy family and with strong personal relationships with political and academic actors. In the 1840s, Florence sparked her interest in public health, a time when a health reform was being debated in the UK as a whole. The Nursing labor was part of her public health interests, including architecture and urban sanitation^(^ 1 ^)^.

At that time, the incredible expansion of production promoted by the Industrial Revolution increased the intensity of production and caused a relative reduction in the amount invested in the purchase of labor power available on the market by free workers^(^ 2 ^)^. Thus, the workers who left the countryside for the city in search of wage labor found themselves in the need to subject themselves to taking on unhealthy jobs and forcing their children into equally degrading situations, such as in the coal mines and in the workhouses^(^ 2 ^)^.

Against her familys wishes, Florence Nightingale increased her interest in Nursing, hospital organization and architecture, and in epidemiological records. She worked in health services in England and, in 1849, visited Nursing services, among other locations, in Rome, Alexandria, Kaiserswerth and Paris. In 1851, she returned to Kaiserswerth for observations and a three-month training in the Kaiserswerther Diakonie and headed to Paris, where she completed her observations and training with the Filles de la charit de Saint-Vincent-de-Paul. Returning to England in 1853, she assumed the position of superintendent at the Establishment for Gentlewomen During Temporary Illness until October 1854, when she embarked for the Ottoman Empire to act alongside the British troops as superintendent of the Female Nursing Establishment of the English General Hospitals in Turkey in the Crimean War^(^ 1_)_.

In skdar, at the Barrack Hospital, Florence Nightingale found an environment lacking instruments and supplies for personal and environmental hygiene, for adequate food and for the comfort of wounded and sick combatants^(^ 1 ^)^. After strong resistance from direct superiors to improve the sanitary conditions at the Barrack Hospital, Florence Nightingale participated in the creation of a health commission, headed by physician John Sutherland, who, after arriving in March 1855, and with her support and that of her subordinates, transformed the health conditions of the hospital^(^ 3 ^)^.

Upon returning to England in 1855, Florence was a national heroine for her contributions to reducing mortality at the Barrack Hospital, having received several obeisances. To honor her, her friends came up with the idea of creating a care institution that would be a kind of English Kaiserswerth through a fund, the Nightingale Fund, established in 1857^(^ 1 ^)^. In 1859, with a collection close to 5.7 million in current values, the Nightingale Fund starts the construction of The Nightingale Home and Training School for Nurses at the newly built St Thomas Hospital in London, England. Inaugurated in July 1860, it received its first students and began a journey of deep transformation in world Nursing towards the professionalization of the so-called art of caring.

Today, there is more than one work process in Nursing administrative, teaching and research all of them acting in function of the Nursing labor^(^ 4 ^)^. The Nursing labor, considered regardless of any determined social form, is characterized today by the labor act of caring for human beings that is performed by human beings in the role of Nursing laborers. They apply esoteric knowledge^(^ 5 ^-^ 6 ^)^ and set up interactions with their service users in a given environment.

When a work is subsumed to the capitalist mode of production, it ceases to be just a process of organic metabolism of the human being with nature in order to produce useful effects to be also a conservative, creative and multiplying value activity. The Nursing labor was no different.

Studies carried out on Nursing in Brazil^(^ 7 ^)^ and with nurses and midwives from Australia, New Zealand and the United Kingdom^(^ 8 ^)^ show that Nursing experiences similar situations in several countries, insufficient workdays, underwages and underemployments, showing the need for reflections on Nursing relationships in the world of labor.

Bearing in mind that in 2020 the 200th anniversary of the birth of the creator of modern Nursing is celebrated, many reflections have been made to discuss the current state of the discipline and profession and the necessary strategies to be implemented in order to continue the advances and its expansion, with a better position in the world of labor; therefore, in this list of investments it is necessary to analyze the development and outcomes of the profession within the dominant sociability system in most of the world, capitalism. Thus, this theoretical essay starts from the following research question: What are the relationships between the development of the Nursing labor and capitalism over the 200 years of Florence Nightingale?

That said, the purpose of this essay is to analyze the relationship between the development of the Nursing labor and capitalism over the 200 years of Florence Nightingale.

## Method

This is an original, discursive, logical-reflective and theoretical production based on interpretations of historical facts that represent turning points in capitalism and that determine the ways to perform the Nursing labor, as well as on Marxist theories that assist in the interpretation of these historical facts. It is organized into analytical categories, created from the method of scientific dialectics as described by Karl Marx^(^ 9 ^)^, in which the economic categories function as ideal expressions of historical relations of production and, therefore, correspond to a certain level of development for the productive forces. The exposure method was developed a posteriori of the investigation process. The categories are the following: creation and expansion of the Nightingalean Nursing Teaching System; subsumption of the Nursing labor to capital; imperialism and international health; and the flexibilization of the Nursing labor.

## Results

Key ideas from the four analytical categories of this reflection are presented. In the creation and expansion of the Nightingalean Nursing Teaching System, The Nightingale Home and Training School for Nurses stands out, established in 1860 at St Thomas Hospital. With a model that was based on pedagogically organized scientific content and the mandatory residency of the students in the hospital, it contributed to raising the quality of service provision and to reducing hospital care costs. Quality and prestige contributed to the international expansion of this System.

The subsumption of the Nursing labor to capital is observed when the work process takes the form of a production process, which is constituted, at the same time, by the work process and the valuation process. The 1929 Crisis, which hit central countries, such as the United States of America (USA), affected the Nursing labor that, with the advent of private health insurance, also began to be directly exploited by capital. The production of more value accompanied by an increase in the workday is one of the main characteristics of the formal subsumption of labor to capital. Concomitantly, the application of Taylorism and Fordism in American hospitals has intensified the productivity of the Nursing labor, characteristic of the real subsumption of labor to capital.

The transition from the 19th century to the 20th century is marked by the development of Imperialism, which has had a major impact on international health. The development of the international market has intensified the routes between countries, moving large intra- and international migratory flows. Such flows and population growth in large urban and commercial centers have contributed to the spread of diseases, especially infectious and epidemic, generating the need for responses from the health field to address the financial concerns of large industries. Within this framework, the Rockefeller Foundation stands out for its performance in the health field.

Based on the influence of the Rockefeller Foundations scientific philanthropy actions, the Nursing labor starts to acquire new skills, such as the application of knowledge about the social aspects of nosological events and health education aimed at the prevention of communicable diseases, and to deepen knowledge in dietetics, biology, pharmacology and other basic sciences. As a result of this influence, the Nursing labor, based on specific and medicalizing public health actions, started to assume important functions in maintaining and recovering a sufficient level of health for the working classes so that they could both offer their workforce and act as consumers in the market society, and was used to clean up the image of large corporations in the oil industry and of the US imperialist interests.

The many crises that marked the capitalist system have, throughout its history, led to important changes in the world of labor. Productive restructuring, reduction of social rights, precarious work, fragmentation of the working class, and weakening of the workers claiming forces are aspects to be considered in the debates on the flexibilization of the Nursing labor, which generates important impacts on health care.

## Discussion

The creation of The Nightingale Home and Training School for Nurses on June 24th, 1860, brought important new contributions, such as: the introduction of scientific knowledge discovered by physicians in the teaching of nurses; the sole responsibility of the superintendent (matron) to control the Nursing service; the need for apprentices to reside in the hospital; the payment of a reasonable amount to nurses after the first year of the course; carrying out manual work exclusively by servants; circulation of apprentices among different wards; and observation as the main competence to be developed by the apprentices^(^ 1 ^)^.

The model lasted three years, as in Kaiserswerth. The first year was devoted to theoretical classes and, in the following two years, hospital and home services were performed^(^ 1 ^)^. Working class apprentices were considered to be ordinary apprentices and the costs of maintaining them in the first year of the program were financed by the Nightingale Fund^(^ 1 ^)^. Upon graduation, the so-called nurses were directed to assume regular obedience positions. The apprentices of the bourgeois classes, referred to as lady nurses, paid for their maintenance costs in the first year of the program and, upon graduation, were directed to assume leadership positions on an international scale^(^ 1 ^)^.

In addition to the prestige of adopting an education system associated with the image of a UK heroine, the adoption of the relatively inexpensive labor of apprentices for two years made hospitals of the time interested in the system. The interest in raising the level of the nurses training and decreasing costs of providing hospital services through the adoption of this system crossed the Atlantic Ocean and reached the USA and Canada^(^ 1 ^)^.

In 1873, three schools implemented Nursing education systems based on the Nightingalean System in the USA: The Bellevue Training School in New York City on May 1st; the Connecticut Training School in New Haven on October 1st; and the Boston Training School on November 1st^(^ 1 ^)^. The Nightingalean System came to be known in the USA as the Bellevue System, as it was introduced in the country by that institution^(^ 1 ^)^. The education systems started disconnected from the hospitals but, due to the lack of donations to finance them, they were integrated into hospitals. In Canada, in 1874, the St. Catharines General and Marine Hospital brought two trained nurses and five or six students from England to set up a teaching system that would last three years^(^ 1 ^)^. In 1875, the Montreal General hospital sought help from Florence Nightingale to set up her education system and five nurses were sent from St. Thomas Hospital to establish the school in the institution^(^ 1 ^)^.

The hospitals in these two countries did not have good working conditions for the nurses. After completing their training in the hospitals, the nurses sought to sell their workforce to families who hired them to provide their services within their homes^(^ 10 ^-^ 11 ^)^. With the arrival of the education system in these countries, the time in which students were trained, on average, tripled, keeping hospitals with more employees on low salaries (with a mean of $10 a month in 1874 in the USA) for longer time^(^ 10 ^)^. The result of this equation was a rapid growth in the number of nurse training schools. Between 1900 and 1929, the number of nursing schools in the USA rose from 432 to 1,885^(^ 10 ^)^. In Canada, the number of nurses between 1901 and 1921 rose from 280 to 21,385, including the students^(^ 11 ^)^.

The process of formal subsumption of labor to the capitalist mode of production is characterized by the conversion of the work process into an instrument of the process of capital self-valorization, although this conversion does not necessarily alter the work process itself^(^ 2 ^)^. This process begins to affect the Nursing labor in the USA with the sale of health insurance, which started in the 1920s, gained popularity in the 1930s, helped in the economic recovery of the hospitals, and boosted the capitalization of these services^(^ 10 ^)^. From 1934 to 1938, the Blue Cross, which was selling insurance for hospital care, went from 100,000 to 1.5 million insured clients, and to 22 million in 1946^(^ 10 ^)^. The Nursing labor already served the capital indirectly by improving workers productive capacity, keeping workers and bourgeois healthy or recovering their health. However, from this process of capitalization of the health services, it is also directly exploited by capital.

While in the process of formal subsumption, the work process only becomes a process of self-valorization of capital, in the process of real subsumption, capital acts in the work process, intensifying it and making it more efficient to produce profit^(^ 2 ^)^. It is characteristic of the real subsumption of labor to capital that the conscious application of sciences to the immediate production process in order to produce more relative value^(^ 2 ^)^. Formal subsumption is a precondition for real subsumption of labor in the capitalist mode of production, since it is necessary to accumulate a minimum of capital in order to invest in the development of new methods of work and in the improvement of its means.

In the Nursing labor, the real subsumption of capital happened pari passu to formal subsumption. As soon as the hospital services were being capitalized, the production of absolute surplus value generated by the excessive increase in daily working hours, the technical division of labor, the loss of ownership of the means of production and the production of relative surplus value generated by application of the Taylorist and Fordist models of work management plagued this in a short period of time.

In the 1930s, the American Nurses Association (ANA) defended the application of Taylorism in hospital care and the consequent technical division of the Nursing labor^(^ 10 ^)^. The ANA proposed that hospitals could hire workers without formal qualifications to perform maintenance services, secretaries for administrative tasks and attendants for routine activities, in order to rationalize the work of registered nurses and reduce labor costs applied to simpler tasks^(^ 10 ^)^. In this way, the ANA promoted an intensification of the separation between manual and intellectual work.

Not only was Taylorism applied to Nursing care, Fordism also influenced the structural changes in Nursing care in the 1930s in the USA^(^ 10 ^)^, as well as the hospital architecture^(^ 12 ^)^. During this period, Functional Nursing emerged, which acted as an assembly line with an intense division of activities into small tasks, and time and movement studies were applied to operations performed in the Nursing labor, which led to a significant increase in the working pace on an industrial scale^(^ 10 ^)^.

Under the pretext of an alleged shortage of nursing professionals caused by the Second World War, American hospitals reversed many previously-achieved advances. They put an end to the eight-hour workload limit; reversed the restriction on the creation of new Nursing schools; they exerted pressure on the War Nursing Council to release the training of 50,000 nurses a year and on the National League for Nursing to loosen the requirements for admission to the Nursing courses, so as to reduce the training time for Nursing students, reintroduce Nursing students to conduct direct assistance in wards and regulate the position of the Licensed Practical Nurse, trained from a one-year technical program^(^ 10 ^)^. Although thousands of nurses left their countries to take part in the war, they represented a small number of the available nurses and governments and private hospitals refused entry to male nurses, black-skinned nurses and people over 40 years old to take up positions in hospitals^(^ 10 ^)^.

Imperialism is a phase of the development of capitalism that started at the turn of the 19th to the 20th century. It is characterized by the acceleration of capital concentration and centralization processes; by the combination; financial capital; inherently capitalist exploitation in the dependence relationships between financial monopolies located in metropolises and their colonies/areas of influence; transformation of the old sharing of the world into sharing between capitalist monopolies over areas of exploitation of raw materials, trade routes and consumer markets; and submission of the international division of labor to the monopolistic interests of the metropolises, under penalty even for using the armed forces^(^ 13 ^)^.

The technologies of the First Industrial Revolution and imperialist interests deeply expanded the production capacity and demanded a greater supply of raw materials, labor instruments, workers and consumer markets^(^ 13 ^)^. In this way, means of land and sea transportation, as well as commercial routes, were developed to move goods and workers to meet the demands of production and capital accumulation, generating large intra- and international migratory flows and eventual contacts for the purchase and sale of goods. In addition, the First World War imposed the movement of large troops in foreign lands. Such elements, combined with the growing population concentration in urban centers, contributed to the population being exposed to microorganisms, for them new, and for the rapid spread of communicable diseases^(^ 14 ^)^.

In the meantime, potential or actual epidemics in one part of the globe came to affect the profits of large international corporations headquartered in other locations^(^ 15 ^)^. Therefore, general health conditions, as well as basic levels of education and the lifestyle/consumption pattern of people from all nations became financial concerns for large industries and philanthropic organizations such as the Carnegie Corporation, created in 1911, the Rockefeller Foundation, created in 1913, and the Ford Foundation, created in 1936, which played an important role in creating conditions favorable to US imperialist exploitation^(^ 15 ^)^.

The Rockefeller familys investment in health-related scientific philanthropy started in 1909, with the Rockefeller Sanitary Commission for the Eradication of Hookworm in Southern USA, followed in 1910 by the Sanitary Commission, until the creation of the Rockefeller Foundation in 1913^(^ 16 ^)^. The Rockefeller Foundation in its creation had the International Health Commission (1913-1916), later named as the International Health Directorate (1916-1927) and, finally, as the International Health Division (1927-1951)^(^ 16 ^)^.

Until 1950, the Rockefeller Foundation had invested or intended to invest in Nursing education programs in almost 30 countries around the world, in addition to promoting the establishment of connections between Nursing schools and universities and proposing the curricular bases of dozens of Nursing education institutions around the world based on the Anglo-American version of the Nightingalean Nursing Teaching System^(^ 17 ^)^, replacing or diminishing the influence of religious Nursing assistance and existing traditional practices where the Foundation had advanced^(^ 16 ^)^. In Brazil, the Rockefeller Foundation played a fundamental role in Nursing and Public Health as a whole, creating schools for the training of nurses and health visitors throughout the national territory^(^ 18 ^)^ and, subsequently, the Special Public Health Service^(^ 19 ^)^.

Crises are the natural mode for the existence of capitalism and, therefore, since its initial stage of development at the international level, the capitalist mode of production has faced several crises^(^ 20 ^)^. The so-called cyclical crises, which took place every 7 or 10 years, occurred within longer periods, around 50 years, which are called long waves^(^ 21 ^)^.

Until the 1950s, there were four long waves in the history of capitalism: (i) between the end of the 18th century and the 1847 crisis; (ii) between 1847 and the beginning of the 1890s; (iii) between the 1890s and the Second World War; and (iv) the last one that started in the mid-1940s^(^ 20 ^)^. Each was initiated from a technological or industrial revolution and all were composed of two phases: an initial expansion, marked by accelerated capital accumulation, and a final one with a tendency to stagnation, marked by gradually decelerated accumulation^(^ 20 ^)^.

The stagnation phase of this last long wave, called the Golden Age of Capitalism, was marked by recessions in imperialist economies in western Europe (capitalist) and Japan between the 1960s and 1970s that culminated in the 1974/75 world recession^(^ 20 ^)^. This phase was also marked by the end of the Bretton Woods Agreement in 1971, the oil crisis caused by the Arab-Israeli conflict in 1973, the neoliberal laboratory installed in the military coup in Chile in 1973 and the crash of the Stock Exchange in 1973/74^(^ 20 ^)^. This period of crisis and recession on a global scale led to the deep deterioration of the Social Welfare State in countries that assumed a central position in the international labor division. In Brazil, contradictorily and due to its quality as a country with a dependent economy, the Unified Health System is built as an element of a universal social security system in the midst of this international setting and, consequently, suffers from pro-market reforms, underfunding and deepening competition between public and private institutions since its inception^(^ 22 ^)^. The 1970s were crucial for capitalism, marking the end of the Social Welfare State guided by Keynesianism, starting the implementation of neoliberal rationality, and marked the beginning of a crisis of another nature: the structural crisis of capitalism^(^ 20 ^)^.

Capital has three fundamental dimensions: production, distribution/circulation/materialization, and consumption. These dimensions, although they have relative autonomy from each other, are fundamentally interdependent and tended to be strengthened, expanded and made increasingly complex through their interactions. When one of these dimensions met a limit, another compensated and gave the feeling that this contradiction had been overcome. With the structural crisis, this triad that leads to the self-expansion of capitals suffers more and more disturbances to the point of presenting flaws in its mechanisms for displacing contradictions from one dimension to another^(^ 20 ^)^.

Unlike the long waves that happened until the 1970s, with the structural crisis there is a situation of cumulative, endemic and chronic crisis on a global scale^(^ 23 ^)^. A productive restructuring was necessary to generate a more flexible pattern of accumulation and to be better able to overcome the contradictions within this crisis^(^ 23 ^)^. This restructuring, which is still under development until the present day, had the Toyota Production System as its main reference^(^ 24 ^)^.

In 1950, Eiji Toyoda visited Fords Rouge plant in Detroit to study the plant and its modes of production and management^(^ 24 ^)^. Upon returning and discussing what he had found in Detroit with Taiichi Ohno, they understood that Fordism would not serve Japan and developed an alternative that would be called the Toyota Production System and, later on, Lean Production^(^ 24 ^)^.

One of the main features of Lean Production is the search for eliminating wastes through processes that promote continuous improvement of the capital accumulation process^(^ 24 ^)^. In Lean Production, wastes are results of operations that do not add value to the product developed^(^ 25 ^)^. To eliminate wastes in the Lean Production, therefore, it is necessary to define what is and what is not value according to the view of its consumer be it the final consumer or some intermediary in the production chain , trace the value chain, that is, define the set of absolutely necessary actions for the production of a certain product or service, and ensure that the entire production process of the product or service reaches the necessary fluidity to meet the needs and desires of the consumers in less time, with fewer errors and with the lowest competitive price^(^ 24 ^-^ 25 ^)^.

With the transformations generated by Lean Production, the intellect of the workers, previously rejected, started to function as an intellect of capital, based on the valorization of value and on the devaluation of work^(^ 26 ^)^. Thus, unproductive work performed by assistants, such as maintenance, monitoring and quality control, are now performed by productive workers^(^ 26 ^)^. Such modifications in the production process generated negative repercussions for social rights, precarious working conditions, greater fragmentation of the working class, and weakening of confrontational unionism to the detriment of negotiating unionism^(^ 26 ^)^.

Lean Production has been applied not only in the automotive industry, but also in other branches of the manufacturing industry and in branches of the service industry, including health care, where it takes the shape of Lean Health Care. Currently, Lean Health Care is not the sole management model that aims to restructure the health industry towards greater flexibilization of work and employment contracts, but many health organizations and systems apply Lean Health Care principles around the world. Some examples with higher prominence are the British National Health System (NHS)^(^ 27 ^)^; the Virginia Mason Medical Center in Seattle, Washington, USA^(^ 28 ^)^, an institution associated to the Virginia Mason Institute, that created the Virginia Mason Production System inspired in Lean Production and makes consultations with diverse health institutions, among them the NHS itself; ThedaCare in Wisconsin, USA, that created the ThedaCare Improvement System based on Lean Production^(^ 29 ^)^; the Institute for Healthcare Improvement in Cambridge, Massachusetts, USA^(^ 30 ^)^; in addition to organizations in Brazil, Canada, Taiwan, New Zealand, Germany, Sweden, Italy and Holland^(^ 29 ^,(^ 31 ^-^ 32 ^)^.

A very comprehensive and promising operational definition was developed for Lean Health Care^(^ 25 ^)^. The authors defined Lean management in health care as a model that integrates the Lean philosophy into the policies and guidelines of health organizations, which is evidenced by the incorporation of Lean principles (such as elimination of waste; continuous improvement of the flow of patients, professionals and supplies; assurance that all processes generate value for consumers; and understanding that frontline workers are the most appropriate to identify and find solutions to everyday factual problems) and a mentality of continuous improvement (which presupposes the understanding that the philosophy is for the long-term and therefore is not limited to a single intervention), and that it is expressed by carrying out Lean evaluation activities (such as value chain mapping, spaghetti diagrams, Gemba walkarounds, root cause analysis and rapid process improvement workshops) and Lean improvement activities (such as 5S or the 6S variation which includes safety(33), level production, visual management and standardized operations)^(^ 25 ^)^.

A key concept for applying the Lean Production principles in the health field is waste. The NHS currently recognizes eight health wastes: (i) Defects: rework caused by failed processes or lack of access to correct information in the first attempt; (ii) Waiting: when the individuals cannot perform their work due to the absence of people, equipment and information at the appropriate time; (iii) Transport: unnecessary movement of materials; (iv) Over processing: performing unnecessary stages that do not add value; (v) Inventory: characterized by excess stocked materials and patients waiting to be discharged or waiting for procedures; (vi) Motion: when people move unnecessarily and for things that are difficult to access; (vii) Over production: the act of producing more than necessary or when it is not necessary; and (viii) Skills: not making the best use of peoples abilities^(^ 34 ^)^.

The Lean Health Care model has been applied in several types of health services and units, such as: emergency^(^ 35 ^-^ 36 ^)^, intensive care units^(^ 35 ^,^ 37 ^-^ 38 ^)^ and coronary units^(^ 37 ^)^, outpatient clinics^(^ 39 ^)^, surgery centers^(^ 35 ^,^ 38 ^-^ 39 ^)^, laboratories^(^ 35 ^,^ 37 ^)^, diagnosis services^(^ 35 ^,^ 39 ^-^ 40 ^)^, pharmaceutical services^(^ 40 ^)^, gynecology and obstetrics^(^ 35 ^)^, primary health care^(^ 40 ^)^ and ophthalmology^(^ 41 ^)^. It is common for the Lean Health Care model to be associated with work management instruments from other models, such as Six Sigma^(^ 35 ^,^ 37 ^,^ 41 ^-^ 42 ^)^.

Nursing laborers, mainly in England, have suffered from the so-called zero-hour contracts^(^ 43 ^)^. In this contemporary model of hiring, the employer does not settle a minimum number of hours worked, and can serve both to fill the void left by job abandonment or by medical leave, and for hiring professionals to work from their homes in a situation of flexible demand for health care^(^ 26 ^,^ 43 ^)^.

From 2016 to 2017, the British National Health System recorded a total of approximately 325,000 workers employed in the zero-hour contracts for adult care, representing almost a quarter (24%) of the entire workforce employed in this sector, with the most affected workers being registered nurses, with 55% of the workforce subjected to this precarious hiring regime, and caregivers, with 56% of the workforce^(^ 43 ^)^. This same report points out that the turnover of these workers is higher in all the scenarios^(^ 43 ^)^. In Brazil, there is currently a trend to intensify the precariousness of the health work, either due to the deterioration of the working conditions or of the contractual employment ties^(^ 44 ^)^.

The area still needs to invest more in studies on the increasing precariousness of the Nursing labor but, from what it has produced, wear out and work overload are verified as consequences, with negative impacts on the physical and mental health of the workers^(^ 45 ^)^. In this sense, for the Nursing labor to produce health care for the population, in the light of the Nightingalean ideas, Nursing laborers need decent conditions to safely exercise their professional practices.

## Conclusion

The Nursing labor, which becomes professional under Florence Nightingales influence, has assumed a dialectical relationship with capitalism in the last 200 years, in which it both determines it and is determined by it. New transformations are yet to come. The technologies of the Fourth Industrial Revolution, also known as Industry 4.0, have demonstrated their ability to radically transform the international labor division, including Nursing. Recovering the visionary impetus of the Lady of the Lamp to lay the foundations of the Nursing transformation is a challenge of the agenda.

## References

[1] Donahue MP (2011). Nursing, The Finest Art: An illustrated history.

[2] Marx K, Karl M, Engels F (1994). Chapter Six. Results of the Direct Production
Process. Marx & Engels Collected Works.

[3] Aravind M, Chung KC (2010). Evidence-Based Medicine and Hospital Reform: Tracing Origins Back
to Florence Nightingale. Plast Reconstr Surg.

[4] Tanaka LH, Leite MMJ (2008). The nurses' working process: the view of professors from a public
university. Acta Paul Enferm. [Internet].

[5] Pekkola E, Carvalho T, Siekkinen T, Johansson J-E, Pekkola E, Kivist J, Kohtamki V, Cai Y, Lyytinen A (2018). The sociology of professions and the study of the academic
profession. Theoretical and Methodological Perspectives on Higher Education
Management and Transformation : An advanced reader for PhD students.

[6] Sakamoto ML (2018). Nursing knowledge: A middle ground exploration. Nurs Philos.

[7] Machado MH, Elaine DO, Lemos W, Lacerda WF, Filho WA, Wermelinger M (2016). Mercado de trabalho da enfermagem: aspectos
gerais. Enferm Foco. [Internet].

[8] Bogossian F, Winters-Chang P, Tuckett A "The Pure Hard Slog That Nursing Is...": A Qualitative
Analysis of Nursing Work (2014). J Nurs. Scholarsh.

[9] Marx KH (2017). Misria da Filosofia.

[10] Wagner D (1980). The Proletarianization of Nursing in the United States,
1932-1946. Int J Heal Serv.

[11] Coburn D (1988). The Development of Canadian Nursing: Professionalization and
Proletarianization. Int J Heal Serv.

[12] Ahuja NK (2012). Fordism in the Hospital: Albert Kahn and the Desing of Old Main,
1917-25. J Hist Med Allied Sci.

[13] Lenin VIU (2011). O Imperialismo: etapa superior do capitalismo.

[14] Silva CM (2013). Nelson Rockefeller e a atuao da American International
Association for Economic and Social Development: debates sobre misso e
imperialismo no Brasil, 1946-1961. Hist Cinc Sade-Manguinhos. [Internet].

[15] Arnove R, Pinede N (2007). Revisiting the "Big Three" Foundations. Crit Sociol.

[16] Magalhes RCS (2016). New history of the Rockefeller Foundation: the odyssey of global
health revisited. Hist Cinc Sade-Manguinhos.

[17] The Rockefeller Foundation (1950). Resume of Rockefeller Foudation Public Health Nursing and Nursing
Education Activities. [Internet].

[18] Oguisso T, Freitas GF, Squires A, Bonini BB (2016). Seeding a Profession: The Intersection of the State,
International Interests, and the Early Development of Brazilian
Nursing. Cult Cuid Rev Enfermera Human.

[19] Campos PFS, Carrijo AR (2019). Eminent but nameless: Lydia das Dres Matta and Brazilian nursing
after 1930. Hist Cinc Sade-Manguinhos.

[20] Mszros I (1995). Beyond capital: Towards a theory of transition.

[21] Mandel EE (1975). Late Capitalism.

[22] Paim JS (2019). Universal health systems and the future of the Brazilian Unified
Health System (SUS). Sade Debate.

[23] Antunes RLC (2013). The meanings of work: Essay on the affirmation and negation of
work.

[24] Chiarini A, Baccarani C, Mascherpa V (2018). Lean production, Toyota Production System and Kaizen
philosophy. TQM J.

[25] Rotter T, Plishka C, Lawal A, Harrison L, Sari N, Goodridge D (2019). What Is Lean Management in Health Care? Development of an
Operational Definition for a Cochrane Systematic Review. Eval Health Prof.

[26] Antunes R (2018). The New Service Proletariat. Mon Rev. [Internet].

[27] NHS Improvement (2019). NHS partnership with Virginia Mason Institute. [Internet].

[28] Phillips J, Hebish LJ, Mann S, Ching JM, Blackmore CC (2016). Engaging Frontline Leaders and Staff in Real-Time
Improvement. Jt Comm J Qual Patient Saf.

[29] D'Andreamatteo A, Ianni L, Lega F, Sargiacomo M (2015). Lean in healthcare: A comprehensive review. Health Policy (New York).

[30] Scoville R, Little K (2014). Comparing Lean and Quality Improvement. [Internet].

[31] Siqueira CL, Siqueira FF, Lopes GC, Gonalves MC, Sarantopoulos A (2019). Enteral diet therapy: use of the Lean Healthcare philosophy in
process improvement. Rev Bras Enferm. [Internet].

[32] Zeferino EBB, Sarantopoulos A, Spagnol GS, Min LL, Freitas MIP (2019). Value Flow Map: application and results in the disinfection
center. Rev Bras Enferm. [Internet].

[33] Fillingham D (2007). Can lean save lives. Leadersh Health Serv (Bradf Engl).

[34] NHS Improvement (2018). Lean - Ohno's eight wastes. [Internet].

[35] DelliFraine JL, Langabeer JR, Nembhard IM (2010). Assessing the Evidence of Six Sigma and Lean in the Health Care
Industry. Qual Manag Health Care.

[36] Holden RJ (2011). Lean Thinking in Emergency Departments: A Critical
Review. Ann Emerg Med.

[37] Glasgow JM, Scott-Caziewell JR, Kaboli PJ (2010). Guiding Inpatient Quality Improvement: A Systematic Review of
Lean and Six Sigma. Jt Comm J Qual Patient Saf.

[38] Nicolay CR, Purkayastha S, Greenhalgh A, Benn J, Chaturvedi S, Phillips N (2012). Systematic review of the application of quality improvement
methodologies from the manufacturing industry to surgical
healthcare. Br J Surg.

[39] Vest JR, Gamm LD (2009). A critical review of the research literature on Six Sigma, Lean
and StuderGroup's Hardwiring Excellence in the United States: the need to
demonstrate and communicate the effectiveness of transformation strategies
in healthcare. Implement Sci.

[40] Mazzocato P, Savage C, Brommels M, Aronsson H, Thor J (2010). Lean thinking in healthcare: a realist review of the
literature. BMJ Qual Saf.

[41] Sommer AC, Blumenthal EZ (2019). Implementation of Lean and Six Sigma principles in ophthalmology
for improving quality of care and patient flow. Surv Ophthalmol.

[42] Deblois S, Lepanto L (2016). Lean and Six Sigma in acute care: a systematic review of
reviews. Int J Health Care Qual Assur.

[43] Skills for Care's Workforce Intelligence (2017). The state of the adult social care sector and workforce in England, 2017
[Internet].

[44] Souza HS, Mendes AN (2016). Outsourcing and "dismantling" of steady jobs at
hospitals. Rev Esc Enferm USP.

[45] Prez EF, David HMSL (2019). Trabalho de Enfermagem e Precarizao: uma Reviso
Integrativa. Enferm em Foco.

